# CC16 Regulates Inflammation, ROS Generation and Apoptosis in Bronchial Epithelial Cells during *Klebsiella pneumoniae* Infection

**DOI:** 10.3390/ijms222111459

**Published:** 2021-10-24

**Authors:** Sultan Almuntashiri, Yohan Han, Yin Zhu, Saugata Dutta, Sara Niazi, Xiaoyun Wang, Budder Siddiqui, Duo Zhang

**Affiliations:** 1 Clinical and Experimental Therapeutics, College of Pharmacy, University of Georgia and Charlie Norwood VA Medical Center, Augusta, GA 30912, USA; salmuntashiri@augusta.edu (S.A.); yohan@augusta.edu (Y.H.); yinzhu@augusta.edu (Y.Z.); saugata.dutta@uga.edu (S.D.); xiaoyunwang@uga.edu (X.W.); 2 Department of Clinical Pharmacy, College of Pharmacy, University of Hail, Hail 55473, Saudi Arabia; 3 College of Pharmacy, University of Georgia, Augusta, GA 30912, USA; sara.niazi25@uga.edu; 4 Division of Infectious Diseases, Medical College of Georgia, Augusta University, Augusta, GA 30912, USA; bsiddiqui@augusta.edu; 5 Vascular Biology Center, Augusta University, Augusta, GA 30912, USA

**Keywords:** SCGB1A1, bacterial pneumonia, lung injury, cell death, innate immunity, NF-κB pathway

## Abstract

Gram-negative (G-) bacteria are the leading cause of hospital-acquired pneumonia in the United States. The devastating damage caused by G- bacteria results from the imbalance of bactericidal effects and overwhelming inflammation. Despite decades of research, the underlying mechanisms by which runaway inflammation is developed remain incompletely understood. Clara Cell Protein 16 (CC16), also known as uteroglobin, is the major protein secreted by Clara cells and the most abundant protein in bronchoalveolar lavage fluid (BALF). However, the regulation and functions of CC16 during G- bacterial infection are unknown. In this study, we aimed to assess the regulation of CC16 in response to *Klebsiella pneumoniae* (*K. pneu*) and to investigate the role of CC16 in bronchial epithelial cells. After *K. pneu* infection, we found that CC16 mRNA expression was significantly decreased in bronchial epithelial cells. Our data also showed that *K. pneu* infection upregulated cytokine and chemokine genes, including IL-1β, IL-6, and IL-8 in BEAS-2B cells. Endogenously overexpressed CC16 in BEAS-2B cells provided an anti-inflammatory effect by reducing these markers. We also observed that endogenous CC16 can repress NF-κB reporter activity. In contrast, the recombinant CC16 (rCC16) did not show an anti-inflammatory effect in *K. pneu-*infected cells or suppression of NF-κB promoter activity. Moreover, the overexpression of CC16 reduced reactive oxygen species (ROS) levels and protected BEAS-2B cells from *K. pneu*-induced apoptosis.

## 1. Introduction

Pneumonia, in general, occurs due to airborne infections caused by bacteria, viruses, fungi, and parasites. There are four types of pneumonia: community-acquired (CAP), hospital-acquired (HAP), healthcare-associated (HCAP), and ventilator-associated pneumonia (VAP) [1]. It is the eighth leading cause of death in the United States and the foremost cause of death from infectious diseases in adults >65 years old, contributing significant morbidity and mortality [2]. HAP is also related to high morbidity and mortality with high healthcare cost. In particular, about 40% of HAP is responsible for the intensive care unit (ICU), and out of these HAPs, up to 90% are VAP. Although HCAP can be included in HAP and CAP, it can be separated from HAP and CAP due to the requirement of treatment for multidrug-resistant pathogens [3].

G- bacteria are among the most serious public health issues in the world. These microorganisms are responsible for the increased resistance to antibiotic treatments, resulting in treatment failure, higher hospital costs, morbidity, and mortality [4]. In addition, the mortality rate is much higher in G- infections than in Gram-positive infections [5]. Among the G- bacteria, *Escherichia coli* is the most common G- microorganism. Other G- pathogens that are responsible for infections are *Klebsiella* spp., *Pseudomonas* spp., *Enterobacter* spp., *Acinetobacter* spp., and et al. [6]. *K.pneu* is a G- bacterium that can cause serious healthcare-associated infections, including pneumonia and urinary tract infections as well as bloodstream infections [7]. It is the leading cause of HAP and VAP, accounting for about 10% of these cases [8]. It is also associated with the increasing rate of multidrug-resistant strains worldwide [9].

CC16 is also known as club cell secretory protein or uteroglobin. It is a 10–16 kDa protein originally secreted by the non-ciliated bronchial epithelial cells in the respiratory epithelium and can also be easily detected in the circulatory system [10]. This protein appears to have a protective effect against respiratory inflammatory responses [11], and it has been studied as a biomarker for lung epithelial injury in most pulmonary diseases, including chronic obstructive pulmonary disease (COPD), asthma, idiopathic pulmonary fibrosis (IPF), acute respiratory distress syndrome (ARDS), sarcoidosis, and pulmonary infections [12,13,14,15,16,17,18,19,20,21,22,23]. As a treatment, CC16 has been tested in both clinical and experimental studies for different lung diseases, such as pulmonary fibrosis, COPD, and respiratory distress syndrome [24,25,26]. In clinical trials, rCC16 was evaluated as promising therapy for respiratory distress syndrome in preterm infants [27]. However, the expression profiles of CC16 in response to bacterial pneumonia as well as the therapeutic effects of CC16 in bacterial infections have not been studied yet. Thus, in the current study, we aimed to investigate the regulation of CC16 in response to *K. pneu* and to evaluate the effects of CC16 in *K. pneu*-infected bronchial epithelial cells.

## 2. Results

### 2.1. K. pneu Infection Decreases CC16 Expression in Bronchial Epithelial Cells and Increases Proinflammatory Cytokines

**Figure 1:**
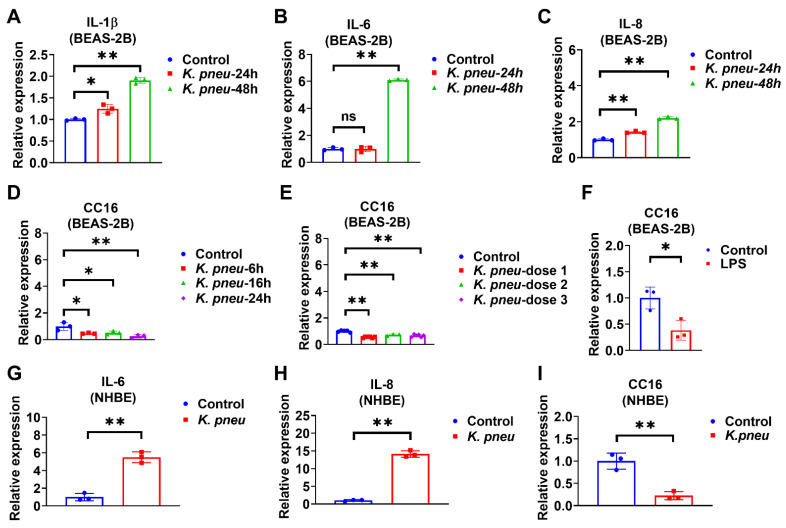
*K. pneu* infection decreases CC16 expression in bronchial epithelial cells and increases the proinflammatory cytokines at different time points. The mRNA levels of proinflammatory cytokines and CC16 were detected from human lung epithelial cells, BEAS-2B cells, which were infected with a cell: *K. pneu* ratio of 1:5 for one hour and then BEAS-2B cells were incubated with 50 µg/mL of gentamycin for the indicated time before mRNA levels were measured (**A**–**D**). mRNA expression levels of IL-1β (**A**), IL-6 (**B**), and IL-8 (**C**) after *K. pneu* infection in BEAS-2B for 24 h and 48 h. (**D**) CC16 mRNA expression levels after *K. pneu* infection in BEAS-2B cells for 6 h, 16, and 24 h. (**E**) CC16 mRNA expression levels in response to a different amount of *K. pneu* infection; dose 1; a cell: *K. pneu* ratio of 1:5, dose 2; a cell: *K. pneu* ratio of 1:10, dose 3; a cell: *K. pneu* ratio of 1:15. (**F**) CC16 mRNA expression levels after treating 200 ng/mL of LPS treatment for 24 h. (**G**–**I**) NHBE cells were infected with a cell: *K. pneu* ratio of 1:5 for one hour. After removing the bacteria, NHBE cells were cultured for 24 h with 50 µg/mL of gentamycin. The mRNA expression levels of IL-6 (**G**), IL-8 (**H**), and CC16 (**I**) were measured using real-time qPCR. Results represent mean ± SD of 3 independent experiments. * *p* < 0.05, ** *p* < 0.01.

Bronchial epithelial cells are located at the interface of the environment and the organism with critical functions, including barrier protection, fluid balance, and mucus and surfactant production [28]. In addition, epithelial cells can initiate immune responses against pathogens by secreting cytokines [29]. Firstly, BEAS-2B cells were infected with *K. pneu,* and our data showed that pro-inflammatory markers, including IL-1β, IL-6, and IL-8, significantly increased (Figure 1A–C). Next, CC16 mRNA expression levels were detected in BEAS-2B cells in response to *K. pneu* infection. We found that CC16 expression significantly decreased in BEAS-2B cells at various time points (Figure 1D). Similarly, reduced CC16 expression was observed in response to different doses of *K. pneu* (Figure 1E). Moreover, CC16 expression also decreased in response to LPS in BEAS-2B cells (Figure 1F). In addition to the cell line, primary Normal Human Bronchial Epithelial Cells (NHBE) were used to validate our observation. *K. pneu* infection consistently induced IL-6 and IL-8 (Figure 1G,H) but was noted to significantly reduce CC16 expression (Figure 1I).

### 2.2. rCC16 Protein Does Not Reduce K. pneu-Induced Inflammation

**Figure 2:**
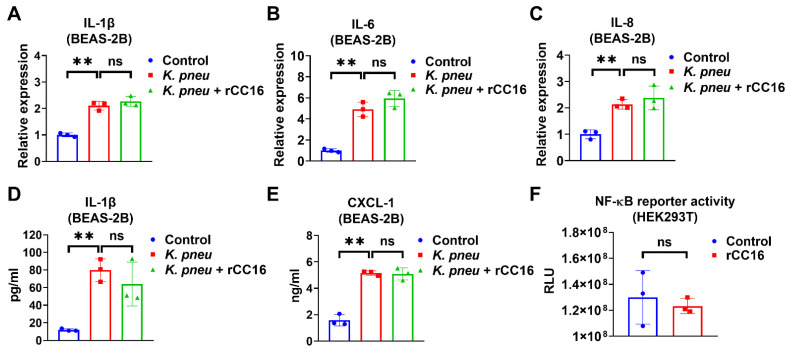
CC16 recombinant protein does not reduce *K. pneu-*induced inflammation. (**A**–**E**) BEAS-2B cells were infected with a 1:5 ratio of *K. pneu* for one hour. BEAS-2B was treated with 50 µg/mL of gentamycin for 48 h with or without 5 μg/mL of CC16 recombinant protein. The mRNA levels of IL-1β (**A**), IL-6 (**B**), and IL-8 (**C**) were determined by real-time polymerase chain reaction (real-time PCR). The secretions of IL-1β (**D**) and CXCL-1 (**E**) were measured using ELISA. (**F**) NF-κB reporter activity was analyzed after treating 200 ng/mL of LPS for 24 h with or without 5 μg/mL of rCC16 treatment. Results represent mean ± SD of 3 independent experiments. ns *p* > 0.05, ** *p* < 0.01.

We evaluated the anti-inflammatory effect of CC16 and observed that 5 μg/mL rCC16 was not able to reduce *K. pneu*-induced IL-1β, IL-6, and IL-8 in BEAS-2B at mRNA level or the release of IL-1β and CXCL-2 from BEAS-2B (Figure 2A–E). However, it was previously reported that rCC16 at doses 2 and 5 μg/mL significantly lowered the expressions of such cytokines in macrophages treated with silica and LPS, respectively [30,31]. Moreover, rCC16 treatment did not change the NF-κB reporter activity in the presence of LPS (Figure 2F). These results indicate that rCC16 at the concentration of 5 μg/mL has no obvious anti-inflammatory effects in bronchial epithelial cells during *K. pneu* infection.

### 2.3. Development of Stable BEAS-2B Cells Overexpressing CC16

**Figure 3:**
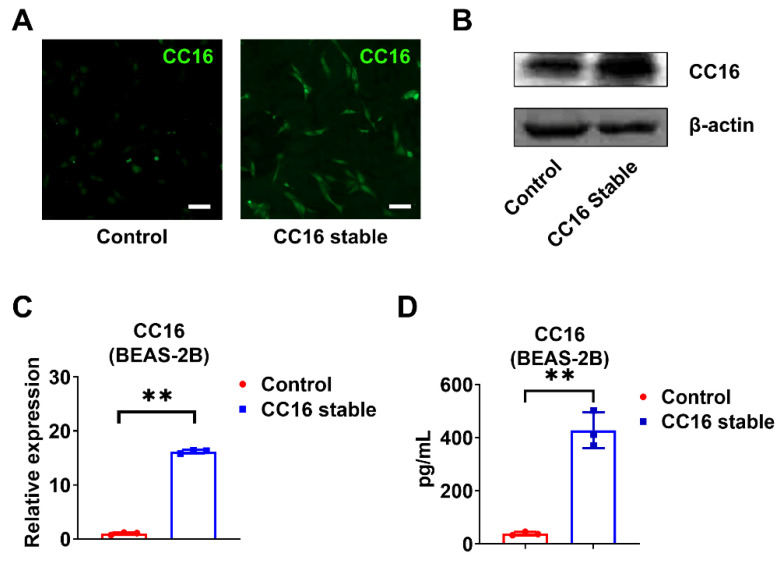
Development of stable BEAS-2B cells overexpressing CC16. (**A**) Immunofluorescence staining images of CC16 were captured in control BEAS-2B and BEAS-2B-CC16. (**B**) The protein expression of CC16 was analyzed by Western blot. (**C**) The mRNA levels of CC16 in BEAS-2B and BEAS-2B-CC16 were measured by real-time PCR. (**D**) The protein levels of CC16 in BEAS-2B and BEAS-2B-CC16 were analyzed by ELISA. Scale bars, 100 µm. Results represent mean ± SD of 3 independent experiments. ** *p* < 0.01.

In order to better understand the role of CC16, we generated CC16 stably overexpressed BEAS-2B cells (BEAS-2B-CC16) and controlled BEAS-2B cells using pRP-Puro-CMV-CC16 and its control vector pRP-Puro-CMV, respectively. The stably transfected cells were selected by puromycin and validated by immunofluorescence staining (Figure 3A) and Western blotting (Figure 3B). CC16 was highly expressed in BEAS-2B-CC16 compared to control BEAS-2B cells in the absence of infection at both mRNA (Figure 3C) and protein levels (Figure 3D).

### 2.4. Overexpression of CC16 Decreases Inflammation, ROS, and Apoptosis during K. pneu Infection

**Figure 4:**
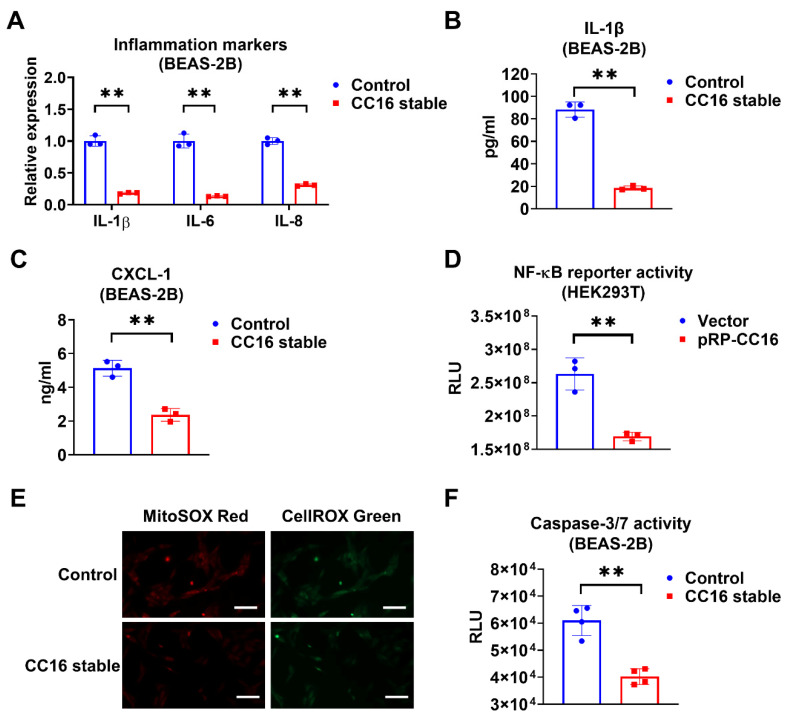
Overexpression of CC16 decreases inflammation, ROS, and apoptosis during *K. pneu* infection. (**A**–**C**) BEAS-2B and BEAS-2B-CC16 were infected with a 1:5 ratio of *K. pneu* for one hour and then incubated in 50 µg/mL of gentamycin for 48 h. The mRNA levels of proinflammatory cytokines IL-1β, IL-6, and IL-8 were determined by real-time PCR (**A**). The secretions of IL-1β (**B**) and CXCL-1 (**C**) were measured using ELISA. (**D**) NF-κB reporter activity in HEK293T cells was measured 24 h after 200 ng/mL of LPS treatment. (**E**,**F**) BEAS-2B and BEAS-2B-CC16 were infected with a 1:5 ratio of *K. pneu* for one hour and then incubated in 50 µg/mL of gentamycin for 48 h. Cells were stained with MitoSOX and CellROX to detect the mitochondrial and cellular oxidative stress levels. Scale bars = 100 µm (**E**). The activities of Caspase 3/7 were measured after infection (**F**). Results represent mean ± SD of 3 independent experiments. ** *p* < 0.01.

Next, we compared the inflammatory responses in BEAS-2B-CC16 and control BEAS-2B cells after *K. pneu* infection. We found that IL-1β, IL-6, and IL-8 expressions were significantly reduced in BEAS-2B stable CC16 cells, whereas control BEAS-2B appeared to have higher mRNA levels of proinflammatory cytokines (Figure 4A). Additionally, compared with control media, lower levels of IL-1β and CXCL-1 were found in the BEAS-2B-CC16 culture media (Figure 4B,C). These results indicate that endogenous CC16 can effectively lower the inflammatory response to bacterial infection. Furthermore, we performed an NF-κB reporter assay. Consistently, NF-κB promoter activity decreased in HEK293T cells after transfection of the CC16 plasmid (Figure 4D). In addition to analyzing the inflammatory responses in BEAS-2B-CC16 and control BEAS-2B cells, we also compared the ROS levels and Caspase 3/7 activities between these two groups of cells. In the immune system, ROS has an important role in maintaining redox balance and activation of various cellular signaling pathways. The excess accumulation of ROS in the cell could cause damage to cell components, which resulted in cell death processes such as apoptosis [32]. As shown in Figure 4E, endogenously overexpressed CC16 can suppress both cellular and mitochondrial ROS generation in BEAS-2B cells during *K. pneu* infection. Meanwhile, overexpression of CC16 attenuates *K. pneu*-induced apoptosis by reducing the activity of Caspase 3/7 in BEAS-2B cells (Figure 4F).

## 3. Discussion

For several decades, CC16 as a circulating biomarker for lung diseases has been extensively investigated [17]. In the majority of studies, CC16 levels were found lower in asthma and COPD. However, CC16 levels in serum were higher in ARDS, pulmonary fibrosis, and sarcoidosis [12,13,14,15,16,17,18,19,20,21,22,23]. The bronchial tubes (bronchi and bronchioles) are predominantly inflamed in asthma and COPD [33], and it has been previously reported that Clara cells are mainly located in bronchial tubes [34]. This is a potential explanation for reduced CC16 in these clinical conditions, as well as for our experimental observations in BEAS-2B and NHBE cells. In contrast, the alveolar epithelium and the tissues around and between the alveoli are mainly affected by pulmonary fibrosis and pulmonary sarcoidosis [35,36]. Similarly, ARDS is characterized by acute inflammatory damage with respect to the alveolar capillary barrier causing an increase in vascular permeability [37]. In ARDS, pulmonary fibrosis, and sarcoidosis, the large and small airways are not directly injured as they are in asthma and COPD. Furthermore, it has been previously reported that Clara cells have a role in repairing damaged epithelium [38]. They are involved in wound repair and become activated after alveolar injury. In injury remodeling, previous studies have reported that Clara cells can migrate and replace injured alveoli in the lung [39,40,41]. Due to the increased blood capillary permeability of the alveoli in pulmonary fibrosis, in the later stage of sarcoidosis and ARDS, CC16 protein released from migratory Clara cells might be easily diffused into circulation. These are possible explanations for the previous findings of clinical and experimental studies [17]. In pulmonary infections, CC16 has been measured as a lung-specific biomarker in RSV [23] and currently in COVID-19; however, there is no study in the literature aimed at assessing CC16 in response to bacterial infections. To the best of our knowledge, our study is the first to confirm the reduction in CC16 mRNA expression levels in lung epithelial cells after bacterial infection.

In pneumonia, the initiation, continuation, and resolution of inflammation depend on the challenging network of pro-inflammatory and anti-inflammatory cytokines. Cytokines are essential for immunity; however, if their activities are overwhelming, they will cause sepsis and result in multiorgan damage [42]. Thus, controlling the level of pro-inflammatory cytokines is one of the strategies for treating infectious diseases. Previous clinical studies reported that the circulating levels of pro-inflammatory cytokines, including tumor necrosis factor-α (TNF-α), IL-6, and IL-8, are generally elevated in patients with pneumonia [43,44,45,46]. A previous study reported that *K. pneu* infection increased COX-2, IL-1β, IL-6, and TNF-α expression in the murine model [47]. Consistently, our results showed that *K. pneu* infection elevated IL-1β, IL-6, and IL-8 in human epithelial cells.

In a murine model of COPD, rCC16 at 5 µg/g bodyweight resolved pathological damage in the lungs and reduced the production of TNF-α, IL-6, and IL-8 in both the serum and BALF after CS exposure [24]. The authors proposed that the suppression of cytokine secretion is mediated by inhibiting the NF-κB pathway in the bronchial epithelium [24]. In a lamb model of infant respiratory distress syndrome, the combination therapy of surfactant and rCC16 lowered IL-8 in serum and IL-6 in the lung tissue more than surfactant therapy alone [48]. Moreover, the anti-inflammatory effect of 2 µg/mL rCC16 protein has been reported in THP-1 macrophages treated with silica [30]. Pang et al. also showed that 5 µg/mL rCC16 inhibits the expressions of TNF-α, IL-6, and IL-8 in LPS-treated RAW264.7 cells via NF-κB and p38 MAPK pathways [31]. In lung epithelial cells, a recent study found that rCC16 protects against LPS-induced apoptosis and inflammatory responses in A549 cells [49]. However, rCC16 was provided in supraphysiological concentrations, which range from 50 to 200 μg/mL. In our study, rCC16 did not modulate IL-1β, IL-6, or IL-8 levels in human lung epithelial cells at 5 μg/mL concentration after *K. pneu* infection. These results also suggest that the internalization of rCC16 may be essential to its cellular function and that a high concentration of CC16 facilitates the internalization via endocytosis. Thus, it explains our observation that a low dose of rCC16 treatment did not provide any anti-inflammatory effects.

In the current study, we generated a stable cell line overexpressing CC16 protein. Our data showed that CC16 overexpression attenuated the inflammation induced by *K. pneu* infection. Meanwhile, reduced NF-κB promoter activity was detected after CC16 overexpression, suggesting that the anti-inflammatory effect may be mediated by the NF-κB signaling pathway. Moreover, the beneficial effects of CC16 overexpression were observed not only in the inflammatory responses but also in oxidative stress and cell death assays. These findings indicate that intracellular CC16, rather than extracellular CC16, protects bronchial epithelial cells against bacterial infection.

Although bronchial epithelial cell line BEAS-2B and primary NHBE were employed, there were no in vivo experiments performed in our study. Considering the differences between in vitro and in vivo, this could be a major limitation of our current work. Moreover, the study largely focused on the regulation and function of CC16 in bronchial epithelial cells. The detailed molecular mechanism was unexplored. Thus, we aim to address these limitations in our future studies.

**Figure 5:**
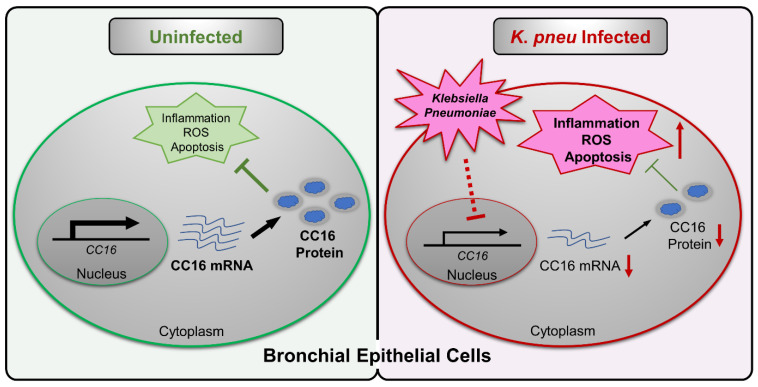
Schematic review of the regulation and function of CC16 in bronchial epithelial cells during *K. pneu* infection. *K. pneu* infection reduces CC16 expression in bronchial epithelial cells. The decreased CC16 level promotes *K. pneu-*induced inflammation, ROS, and apoptosis. Moreover, intracellular rather than extracellular CC16 has an anti-inflammatory effect in bronchial epithelial cells.

In summary, our study suggested intracellular CC16 could serve as an inhibitor of inflammation, ROS, and apoptosis in bronchial epithelial cells (Figure 5). Upon *K. pneu* infection, CC16 expression is significantly downregulated in bronchial epithelial cells, which results in elevated proinflammatory genes expression, such as IL-1β, IL-6, and IL-8. The overexpression of the endogenous CC16 provides a protective effect in bronchial epithelial cells against bacterial infection through the inhibition of inflammation, ROS, and apoptosis. 

## 4. Materials and Methods

### 4.1. Cell Culture 

BEAS-2B and HEK293T cells were purchased from ATCC (Manassas, VA, USA) and cultured in DMEM with 10% FBS and 1% penicillin/streptomycin (GIBCO, Grand Island, NY, USA). Primary NHBE cells were purchased from Lonza Bioscience (Allendale, NJ, USA) and were cultured in BEBM medium (Lonza, Basel, Switzerland) containing BEGM growth supplement (Lonza) until they were confluent. All cells were cultured in an incubator at 37 °C in 5% CO_2_/95% air. 

### 4.2. RNA Isolation and Reverse Transcriptase-Polymerase Chain Reaction

Cells were lysed, and total RNA was extracted by using Trizol reagent (Thermo Fisher Scientific, Waltham, MA, USA). Real-time RT-PCR was performed using the Ultra SYBR Two-Step RT-qPCR Kit (Thermo Fisher Scientific) according to the manufacturer’s instructions. Equal amounts of total RNA (1 μg) from each sample were converted into complementary DNA (cDNA) in a reverse-transcription reaction. PCR for each gene was carried out in a 10 μL reaction mixture containing 1 μL of cDNA template. The expression of the housekeeping gene *TBP* was used as the internal control. The following primers were used: *TBP* forward: 5′-GATAAGAGAGCCACGAACCAC-3′; *TBP* reverse: 5′-CAAGAACTTAGCTGGAAAACCC-3′; *CC10* forward: 5′-AAACCCTCCTCATGGACAC-3′; *CC10* reverse: 5′-TGCTTTCTCTGGGCTTTTG-3′; *IL-1β* forward: 5′-CCACAGACCTTCCAGGAGAATG-3′; *IL-1β* reverse: 5′-GTGCAGTTCAGTGATCGTACAGG-3′; *IL-6* forward: 5′-ACTCACCTCTTCAGAACGAATTG-3′; *IL-6* reverse: 5′-CCATCTTTGGAAGGTTCAGGTTG-3′; *IL-8* forward: 5′-CTGTGTGAAGGTGCAGTTTTGCC-3′; and *IL-8* reverse: 5′-CTCAGCCCTCTTCAAAAACTTCTCC-3′.

### 4.3. Western Blot and ELISA

Western blotting was performed as previously described [50]. In brief, cells were homogenized in RIPA lysis buffer supplemented with Protease Inhibitor Cocktail and Phosphatase Inhibitor Cocktail (Sigma-Aldrich, St. Louis, MO, USA). Antibodies used include CC16 (ab213203), which was purchased from Abcam (Cambridge, MA, USA). Antibodies used include β-actin (A5441), which was purchased from Sigma Chemicals Co. (St. Louis, MO, USA). The DuoSet ELISA Kits for human CC16 (DY4218), IL-1β (DY201), and CXCL-1 (DY275) were purchased from R&D Systems to confirm CC16 protein amount in BEAS-2B and BEAS-2B-CC16 or the concentrations of IL-1β and CXCL-1 in the BEAS-2B cell culture media after treatment according to the manufacturer’s protocol.

### 4.4. Bacterial Count and In Vitro Bacterial Infection

*K. pneu* (ATCC) was cultivated in LB liquid medium (Affymetrix, Inc. OH, USA) to the logarithmic phase at 37 °C while rotating at 250 rpm until the exponential phase of the bacterial growth was reached (OD_600_ = 1). The bacterial culture was diluted in phosphate-buffered saline to a concentration of 1.0 × 10^6^ bacteria/mL. The amounts of 1 × 10^6^ of BEAS-2B and NHBE were seeded in 60 mm cell culture dishes overnight. After culturing overnight, the cells were infected with *K. pneu* at a multiplicity of infection (MOI) of 1:5, 1:10, and 1:15 ratios and incubated for 1 h. Then, the medium was removed and incubated in complete DMEM containing gentamycin 50 µg/mL (RPI, IL, USA) for an additional 24 h or 48 h with or without rCC16 5 μg/mL, as indicated in the figure legends. 

### 4.5. NF-κB Reporter Assay

The plasmid pNL3.2. NF-κB-RE (Promega, Madison, WI, USA) that coded five copies of an NF-κB response element with firefly luciferase was transfected. Transfection of this plasmid was performed by using Lipofectamine 2000 according to the manufacturer’s instructions. At 24 h after transfection, a luciferase assay was performed using the Nano-Glo Luciferase Assay System (Promega) according to the manufacturer’s protocol. Firefly luciferase activities were measured and analyzed after treating 100 ng/mL of LPS for 24 h with or without 5 μg/mL of rCC16 treatment.

### 4.6. Stable Cell Generation

Plasmids pRP-Puro-CMV and pRP-Puro-CMV-CC16 were purchased from VectorBuilder Inc. (Chicago, IL, USA). In order to generate stable cell lines, plasmids were transfected into BEAS-2B. Two days post-transfection, 100 µg/mL of hygromycin B was added to the culture medium in order to select plasmid-containing cells. The hygromycin-added media were changed every 3 days until colonies formed.

### 4.7. Immunofluorescence Staining

Control BEAS-2B and BEAS-2B-CC16 cells were seeded in an 8-well chamber slide (3 × 10^3^ cells per well). One day after *K. pneu* infection, the cells were fixed with 4% formaldehyde and permeabilized with 0.1% Triton X-100. Then, the fixed cells were blocked with 3% BSA and followed by CC16 primary antibodies (ab213203, Abcam) incubation overnight at 4 °C. Next, after incubating with Alexa 488 conjugated secondary antibodies (Thermo Fisher Scientific) for 1 h, the slides were washed with PBS. The images were captured using a Zeiss Observer Z1 microscope (Carl Zeiss, Oberkochen, Germany).

### 4.8. Measurement of Cellular and Mitochondrial ROS Production

Cellular ROS and mitochondrial ROS were measured using the CellROX™ Green Reagent and MitoSOX™ Red Mitochondrial Superoxide Indicator (Invitrogen, Waltham, MA, USA), respectively. BEAS-2B-CC16 amounting at 1 × 10^6^ and its control cells were seeded in 60 mm cell culture dishes and infected by *K. pneu* for 1 h. Then, the cells were incubated in a complete culture medium containing gentamycin for an additional 24 h. CellROXGreen Reagent measuring 5 μM was treated for 30 min at 37 °C, and 5 μM of Mito SOX Red reagent was treated for 10 min at 37 °C. The fluorescence images of the cells were captured by using a Zeiss Observer Z1 microscope (Carl Zeiss).

### 4.9. Determination of Caspase-3/7 Activities

The activities of caspases in cells were measured using the Caspase-Glo 3/7 Assay kit (Promega) according to the manufacturer’s protocol. Briefly, 1 × 10^4^ of cells were seeded in 96-well plates and incubated with or without *K. pneu* as described. Then, Caspase-Glo^®^ 3/7 Reagent was added and incubated for 30 min. The luciferase activities were measured using Promega GloMax^®^ Discover Microplate Reader.

### 4.10. Statistical Analysis

All statistical analyses were conducted using GraphPad Prism software (San Diego, CA, USA). All data were presented as means ± SD. All the data from three independent experiments were averaged before normalization. For real-time qPCR (Thermo Fisher Scientific), the same amount of cDNA was used, and all data were analyzed at the same time. Comparisons between 2 groups were performed using a two-tailed unpaired Student’s t-test. Multiple groups were compared using a one-way ANOVA with the Tukey method. *p*-value of less than 0.05 was considered statistically significant.

## Data Availability

The datasets generated during and/or analyzed during the study are available from the corresponding author upon reasonable request.
